# Editorial: Emerging Zoonoses: Eco-Epidemiology, Involved Mechanisms, and Public Health Implications

**DOI:** 10.3389/fpubh.2015.00157

**Published:** 2015-06-08

**Authors:** Rubén Bueno-Marí, A. Paulo Gouveia Almeida, Juan Carlos Navarro

**Affiliations:** ^1^Entomology and Pest Control Laboratory, Cavanilles Institute of Biodiversity and Evolutionary Biology (ICBiBE), University of Valencia, Valencia, Spain; ^2^Global Health and Tropical Medicine (GHTM), Instituto de Higiene e Medicina Tropical, Universidade Nova de Lisboa, Lisbon, Portugal; ^3^University of Pretoria, Pretoria, South Africa; ^4^Instituto de Zoología y Ecología Tropical, Universidad Central de Venezuela, Caracas, Venezuela

**Keywords:** zoonoses, infectious diseases, infectious diseases epidemiology, editorial, one health

Zoonoses are currently considered as one of the most important threats for Public Health worldwide. Zoonoses can be defined as any disease or infection that is naturally transmissible from vertebrate or invertebrate animals to humans and vice-versa. Approximately, 75% of recently emerging infectious diseases affecting humans are diseases of animal origin; approximately, 60% of all human pathogens are zoonotic. All types of potential pathogenic agents, including viruses, parasites, bacteria, and fungi, can cause these zoonotic infections. From the wide range of potential vectors of zoonoses, arthropods are probably those of major significance due to their abundance, high plasticity, adaptability, and coevolution to different kinds of pathogens, high degrees of synanthropism in several groups, and difficulties to apply effective programs of population control. Although ticks, flies, sandflies, cockroaches, bugs, and fleas are excellent vectors capable of transmitting viruses, parasites, and bacteria, undoubtedly mosquitoes are the most important human disease vectors, while ticks are the most important vectors of pathogens in domestic production animals. Mosquito borne diseases like malaria, equine encephalitis, or West Nile are zoonoses with increasing incidence in the last years in tropical and temperate countries. All these zoonoses are thoroughly discussed in the Research Topic (1–5). Moreover, several researches focused on new tools to fight against Dengue vectors (6), studies about mosquito biodiversity (7), or novel modeling techniques based on climatic factors to predict vector’s incidence (8) can also be found in our compilation of research works related with zoonoses. Although it is well known that mosquitoes are the major vectors worldwide, probably ticks and tick-borne diseases are those that have aroused higher interest in epidemiologists and medical entomologists in recent years (9–12).

The problems related with zoonoses have different significance in developed and undeveloped countries. One example of a vector-borne disease relatively easy to combat with current pharmacological, preventive, and vector control tools but with a dramatic incidence in Central and South America is Chagas disease or American trypanosomiasis (13–19). In Africa and Asia, other neglected diseases like leishmaniasis or African trypanosomiasis have serious impact on human populations locally (20–22).

Not all zoonoses are vector borne, vertebrates can also transmit serious zoonoses, highlighting the role of some carnivorous animals in rabies dissemination, the spread of rodent borne diseases in several rural and urban areas, or some transmissible bacteria in cattle and other livestock (23).

According to WHO, FAO, and OIE guidelines, an emerging zoonotic disease can be defined as a zoonosis that is newly recognized or newly evolved, or that has occurred previously but shows an increase of incidence or expansion in geographical, host, or vector range. There are many factors that can provoke or accelerate the emergence of zoonoses, such as environmental changes, habitat modifications, variations of human and animal demography, pathogens and vectors anomalous mobilization related with human practices and globalization, such as the introduction of exotic mosquito species of which *Aedes albopictus* is the paradigm, deterioration of the strategies of vector control, or changes in pathogen genetics (24–26). To reduce Public Health risks from zoonoses, it is absolutely necessary to acquire an integrative perspective that includes the study of the complexity of interactions among humans, animals, and environment in order to be able to fight against these issues of primary interest for human health, hence the new “One Health” approach. In any case, although zoonoses represent significant public health threats, many of them still remain as neglected diseases and consequently are not prioritized by some national or international health organisms.

The aim of this Research Topic is to cover all related fields with zoonoses, including basic and applied researches, approaches to control measures, explanations of new theories or observations, opinion articles, reviews, etc. To deeply discuss these issues, a holistic and integrative point of view is obviously needed and guided by the “One Health” strategy. Editors are very proud to say that this ambitious goal for the Research Topic has been accomplished, thanks to the collaboration of researchers specialized in different fields as medical and veterinary entomologists, parasitologists, veterinarians, virologists, zoologists, microbiologists, ecologists, evolutionary biologists, and medicals specialized in epidemiology, public health, and animal health. The participation of multiple contributors and a multidisciplinary approach have been most important to comply with a knowledge demand of this issue of first-rate of scientific and medical interest.

## Conflict of Interest Statement

The authors declare that the research was conducted in the absence of any commercial or financial relationships that could be construed as a potential conflict of interest.
